# Supplementary material for: Global stroke burden attributable to household air pollution: Insights from GBD 2021 and projections to 2040
Source: PLoS One. 2025 Jul 29;20(7):e0327976. doi: 10.1371/journal.pone.0327976 (PMC12306745; doi:10.1371/journal.pone.0327976)
Supplement: S1 File — Global Stroke Burden Attributable to Household Air Pollution: Insights from GBD 2021 and Projections to 2040. (DOCX) [file pone.0327976.s001.docx]

**Supplementary Material**

**Global Stroke Burden Attributable to Household Air Pollution: Insights from GBD 2021 and Projections to 2040**

**Supplementary Tables**

**S1 Table.** The numbers and age-standardized rates of deaths, DALYs, YLDs, and YLLs due to household air pollution-related stroke globally in 1990 and 2021, as well as trends from 1990 to 2019.

**S2 Table.** Trends in the burden of disease for males and females from 1990 to 2021.

**S3 Table.** Differences in the burden of disease across regions with different levels of social development from 1990 to 2021: a global comparative study based on SDI stratification.

**S4 Table.** Distribution characteristics of disease burden across 21 global regions.

**S5 Table.** Regional differences in the global burden of disease in 2021: a cross-regional comparative study based on age-standardized rates.

**S6 Table.** Global Trends in Household Air Pollution-Induced Stroke Outcomes: EAPC Analysis Across 204 Countries for Deaths, DALYs, YLDs, and YLLs (1990-2021).

**S7 Table.** Global Burden of Disease Metrics for 1990, 2021, and 2040 (Predicted)

**S1 Table.** The numbers and age-standardized rates of deaths, DALYs, YLDs, and YLLs due to household air pollution-related stroke globally in 1990 and 2021, as well as trends from 1990 to 2019.

| Measure | 1990 | | 2021 | | 1990-2021 |
| --- | --- | --- | --- | --- | --- |
|  | Number (95% UI) | ASR/100000 (95% UI) | Number (95% UI) | ASR/100000 (95% UI) | EAPC (95% CI) |
| Deaths | 594570 (410656-816224) | 17.42 (12.14-23.95) | 1230852 (834767-1575049) | 14.78 (10.05-18.92) | -0.37 (-0.50--0.24) |
| DALYs | 13337927 (9219590-18469720) | 349.70 (241.10-483.15) | 26779430 (18076188-34223093) | 312.85 (211.45-399.79) | -0.20 (-0.34--0.07) |
| YLDs | 881989 (547471- 1316934) | 21.89 (13.56- 32.78) | 2425770 (1536922- 3438131) | 28.22 (17.89- 40.03) | 1.11 (0.94-1.27) |
| YLLs | 12455938 (8545738-17215334) | 327.81 (224.92-449.66) | 24353661 (16319005-31243808) | 284.63 (191.00-364.58) | -0.30 (-0.44--0.17) |

EAPC: Estimated Annual Percentage Change; UI: uncertainty intervals; CI: Confidence Interval; DALYs: Disability-Adjusted Life Years; YLDs: Years Lived with Disability; YLLs: Years of Life Lost.

**S2 Table.** Trends in the burden of disease for males and females from 1990 to 2021.

| Measure | Sex | 1990 | | 2021 | | 1990-2021 |
| --- | --- | --- | --- | --- | --- | --- |
|  |  | Number (95% UI) | ASR/100000 (95% UI) | Number (95% UI) | ASR/100000 (95% UI) | EAPC (95% CI) |
| Deaths | Male | 293037 (197900-414330) | 19.67 (13.36-27.35) | 683976 (4605983-883020) | 18.64 (12.63- 24.03) | 0.06 (-0.12-0.24) |
|  | Female | 301533 (209694-417003) | 15.51 (10.81-21.49) | 546876 (355546-710729) | 11.71(7.61-15.22) | -0.83 (-0.93--0.73) |
| DALYs | Male | 7173708.80 (4792684.40-10211357.36) | 408.20 (275.13-578.09) | 15531548 (10404840-19914852) | 391.97 (263.55-502.98) | 0.08 (-0.09-0.26) |
|  | Female | 6164218.08 (4226821.02-8571371.93) | 297.54 (204.33-412.85) | 11247883 (7258687-14524263) | 244.08 (157.48-315.14) | -0.56 (-0.66--0.45) |
| YLDs | Male | 432059.14 (263304.90-657311.33) | 23.33 (14.14-35.27) | 1223989 (785144-1726152) | 30.31 (19.52-42.71) | 1.17 (1.00-1.33) |
|  | Female | 449929 (276676-660466) | 20.84 (12.81-30.70) | 1201781 (727026-1689392) | 26.46 (16.02-37.21) | 1.02 (0.85-1.19) |
| YLLs | Male | 6741650 (4507675-9493434) | 384.87 (259.63-543.93) | 14307559 (9524061-18547426) | 361.66 (241.74-468.67) | 0.01 (-0.17-0.19) |
|  | Female | 5714289 (3918978-7890359) | 276.70 (190.02-382.56) | 10046102 (6482973-13011274) | 217.62 (140.38-281.89) | -0.70 (-0.81--0.60) |

EAPC: Estimated Annual Percentage Change; UI: uncertainty intervals; CI: Confidence Interval; DALYs: Disability-Adjusted Life Years; YLDs: Years Lived with Disability; YLLs: Years of Life Lost.

**S3 Table.** Differences in the burden of disease across regions with different levels of social development from 1990 to 2021: a global comparative study based on SDI stratification.

| SDI Level | Measure | 1990 | 2021 | 1990-2021 |
| --- | --- | --- | --- | --- |
|  |  | ASR/100000 (95% UI) | ASR/100000 (95% UI) | EAPC (95% CI) |
| High SDI | Deaths | 11.06 (7.21-15.93) | 3.44 (2.56-4.49) | -3.98 (-4.12--3.83) |
|  | DALYs | 219.24 (143.30-314.22) | 79.95 (61.36-101.85) | -3.43 (-3.56--3.30) |
|  | YLDs | 22.69 (13.19-35.29) | 14.66 (9.39-21.20) | -1.54 (-1.66 -1.42) |
|  | YLLs | 196.56 (129.59-278.87) | 65.29 (50.00-83.54) | -3.74 (-3.88--3.61) |
| High-middle SDI | Deaths | 28.57 (19.25-40.18) | 18.46 (13.17-23.70) | -1.36 (-1.61--1.11) |
|  | DALYs | 536.59 (356.23-750.96) | 366.95 (260.97-463.81) | -1.17 (-1.43--0.91) |
|  | YLDs | 30.90 (18.32-45.49) | 37.99 (24.12-52.96) | 1.10 (0.88-1.33) |
|  | YLLs | 505.68 (336.05-711.28) | 328.96 (232.98-418.94) | -1.35 (-1.63--1.07) |
| Middle SDI | Deaths | 18.06 (10.87-27.44) | 23.50 (14.70-30.47) | 1.23 (1.02-1.44) |
|  | DALYs | 376.80 (226.99-573.99) | 472.10 (297.91-602.67) | 1.08 (0.89-1.28) |
|  | YLDs | 19.41 (11.36-31.63) | 38.85 (22.57-54.69) | 2.67 (2.47-2.87) |
|  | YLLs | 357.40 (215.00-543.47) | 433.25 (274.41-556.57) | 0.97 (0.77-1.17) |
| Low-middle SDI | Deaths | 12.04 (7.71-17.40) | 14.81 (8.76-21.12) | 0.90 (0.69- 1.11) |
|  | DALYs | 260.17 (168.78-377.52) | 323.58 (192.02-459.53) | 0.92 (0.71, 1.13) |
|  | YLDs | 12.10 (7.03-19.75) | 19.42 (10.74-28.81) | 1.80 (1.55-2.04) |
|  | YLLs | 248.07 (159.27-358.71) | 304.16 (180.16-435.66) | 0.87 (0.66-1.08) |
| Low SDI | Deaths | 11.03 (7.03-15.96) | 9.75 (6.22-14.32) | -0.05 (-0.36-0.27) |
|  | DALYs | 242.79 (155.20-350.00) | 208.38 (132.39-306.80) | -0.20 (-0.49-0.09) |
|  | YLDs | 11.49 (6.76-17.46) | 12.00 (7.11-18.65) | 0.53 (0.26-0.80) |
|  | YLLs | 231.30 (148.26-333.89) | 196.39 (126.03-287.93) | -0.24 (-0.53-0.05) |

SDI: Socio-demographic Index; EAPC: Estimated Annual Percentage Change; UI: uncertainty intervals; CI: Confidence Interval; DALYs: Disability-Adjusted Life Years; YLDs: Years Lived with Disability; YLLs: Years of Life Lost.

**S4 Table.** Distribution characteristics of disease burden across 21 global regions in 2021.

| Region | Deaths  Number (95% UI) | YLLs  Number (95% UI) | YLDs  Number (95% UI) | DALYs  Number (95% UI) |
| --- | --- | --- | --- | --- |
| **Asia** |  |  |  |  |
| East Asia | 645,929 (413,797-844,249) | 11,925,362 (7,541,071-15,629,812) | 1,263,301(761,113-1,786,008) | 13,188,662.9 (8,474,561.1-17,093,130.2) |
| South Asia | 178,399 (102,441-251,221) | 4,131,663 (2,387,086-5,838,008) | 304,413 (168,087-447,091) | 4,436,075 (2,555,886-6,239,616) |
| Southeast Asia | 114,414 (66,200.2-158,168.7) | 2,637,328 (1,539,663.6-3,672,723.3) | 221,645 (123,805-336,248) | 2,858,972 (1,673,471-3,953,880) |
| Central Asia | 14,266 (9,843-19,110) | 306,255 (207,869-413,185) | 34,809(21,551-49,959) | 341,064 (232,096-454,622) |
| High-income Asia Pacific | 19,857 (11,481-29,945) | 281,925 (164,987-417,264) | 90,072(50,197-138,607) | 372,000 (223,036-547,665) |
| **Europe** |  |  |  |  |
| Eastern Europe | 35,962(22,680-54,382) | 630,957 (397,334-951,302) | 55,326 (31,363-83,157) | 686,283 (432,938-1,029,290) |
| Western Europe | 22,514(15,076-32,090) | 293,915(199,418-418,000) | 65,203(40,414-96,925) | 359,117 (245,060-498,395) |
| Central Europe | 27,824 (20,179-35,587) | 438,495 (317,385-560,570) | 42,697(26,939-59,811) | 481,192 (351,231-610,152) |
| **Americas** |  |  |  |  |
| High-income North America | 6,654 (3,285-11,060) | 104,284 (51,218-170,498) | 30,224 (13,707-52,560) | 134,507 (65,312-218,603) |
| Andean Latin America | 3,717  (2,324-5,604) | 79,895 (49,516-121,808) | 7,320 (4,175-11,100) | 87,215 (55,027-131,850) |
| Central Latin America | 8,903(6,070-12,024) | 182,489 (125,102-245,782) | 18,269 (11,427-26,361) | 200,758 (136,854-267,972) |
| Tropical Latin America | 10,436 (5,929-16,122) | 219,113 (124,310-338,517) | 14,546 (7,784-23,718) | 233,659  (132,435-362,111) |
| Southern Latin America | 4,169 (2,328-6,456) | 74,336 (41,860-114,262) | 13,105 (6,947-21,346) | 87,441  (49,779-135,069) |
| Caribbean | 4,316 (2,198-7,158) | 85,991 (44,060-144,514) | 4,740 (2,168-8,192) | 90,731  (45,890-151,597) |
| **Africa and Middle East** |  |  |  |  |
| North Africa and Middle East | 86,444 (65,264-107,726) | 1,858,183 (1,390,599-2,309,951) | 164,689 (112,523-221,840) | 2,022,872  (1,519,952-2,519,320) |
| Western Sub-Saharan Africa | 26,736 (15,080-41,294) | 627,409 (352,773-969,558) | 55,103 (28,592-88,793) | 682,512 (385,341-1,044,992) |
| Eastern Sub-Saharan Africa | 8,077(5,000-12,257) | 198,235(123,970-300,131) | 14,893 (8,477-23,212) | 213,127 (133,485-320,484) |
| Central Sub-Saharan Africa | 4,056 (2,405-6,144) | 100,886 (59,533-153,450) | 7,414  (4,256-11,421) | 108,300 (64,652-163,472) |
| Southern Sub-Saharan Africa | 6,939 (4,682-9,325) | 153,798 (104,784-204,695) | 14,383 (8,903-20,456) | 168,181 (114,355-223,718) |
| **Oceania** |  |  |  |  |
| Oceania | 507(166-1,148) | 13,433 (4,336.2-30,851.3) | 860 (293-1,915) | 14,294(4,644-32,889) |
| Australasia | 733 (422-1,113) | 9,711 (5,607-14,753) | 2,759 (1,512-4,295) | 12,470 (7,290-18,674) |

UI: uncertainty intervals; DALYs: Disability-Adjusted Life Years; YLDs: Years Lived with Disability; YLLs: Years of Life Lost.

**S5 Table.** Regional differences in the global burden of disease in 2021: a cross-regional comparative study based on age-standardized rates.

| Region | Deaths  ASR/100000 (95% UI) | YLLs  ASR/100000 (95% UI) | YLDs  ASR/100000 (95% UI) | DALYs  ASR/100000 (95% UI) |
| --- | --- | --- | --- | --- |
| **Asia** |  |  |  |  |
| East Asia | 33.00 (21.27-43.00) | 568.77 (361.62-743.17) | 59.03 (35.74-83.78) | 627.80 (404.82-813.83) |
| South Asia | 13.62 (7.80-19.17) | 281.70 (162.19-397.12) | 19.55 (10.80-28.75) | 301.25 (173.06-423.65) |
| Southeast Asia | 20.18 (11.63-27.74) | 408.01 (237.19-565.10) | 32.86 (18.44-49.85) | 440.87 (257.14-607.71) |
| Central Asia | 21.01 (14.56-28.05) | 396.51 (270.92-531.99) | 40.35 (24.96-58.07) | 436.86 (298.64-580.03) |
| High-income Asia Pacific | 3.28 (1.91-4.87) | 59.06 (35.14-86.14) | 21.45 (12.03-33.00) | 80.51 (48.93-118.01) |
| **Africa and Middle East** |  |  |  |  |
| North Africa and Middle East | 23.48 (17.73-29.41) | 431.13 (324.61-536.89) | 32.41 (22.08-43.45) | 463.54 (350.54-575.62) |
| Western Sub-Saharan Africa | 17.19 (9.66-26.51) | 330.90 (186.73-511.13) | 25.66 (13.27-41.13) | 356.56 (201.00-546.19) |
| Southern Sub-Saharan Africa | 14.77 (9.88-20.06) | 278.47 (189.06-372.68) | 25.78 (15.97-36.75) | 304.25 (207.61-407.83) |
| Eastern Sub-Saharan Africa | 5.87 (3.59-8.96) | 118.81 (73.63-180.44) | 8.36 (4.76-12.99) | 127.18 (78.64-192.42) |
| Central Sub-Saharan Africa | 9.91 (5.93-14.81) | 195.64 (116.24-295.33) | 13.08 (7.54-20.02) | 208.73 (124.20-313.94) |
| **Europe** |  |  |  |  |
| Eastern Europe | 10.07 (6.35-15.22) | 181.45 (114.32-273.26) | 16.61 (9.44-24.93) | 198.06 (124.74-296.71) |
| Central Europe | 11.61 (8.42-14.85) | 191.95 (139.00-245.45) | 20.44 (12.93-28.64) | 212.38 (155.05-268.88) |
| Western Europe | 1.86 (1.25-2.65) | 27.82 (19.08-39.36) | 7.14 (4.40-10.61) | 34.96 (23.78-48.45) |
| **Americas** |  |  |  |  |
| Caribbean | 7.92 (4.03-13.16) | 159.75 (81.86-268.77) | 8.87 (4.06-15.33) | 168.62 (85.29-282.18) |
| Andean Latin America | 6.49 (4.07-9.77) | 133.38 (82.69-203.00) | 12.10 (6.89-18.34) | 145.48 (91.95-220.08) |
| Southern Latin America | 4.60 (2.57-7.12) | 85.09 (48.03-130.70) | 15.33 (8.11-24.98) | 100.42 (57.31-155.21) |
| Tropical Latin America | 4.20 (2.39-6.49) | 85.48 (48.49-132.10) | 5.69 (3.04-9.29) | 91.17 (51.66-141.35) |
| Central Latin America | 3.73 (2.54-5.05) | 73.27 (50.19-98.67) | 7.29 (4.56-10.52) | 80.56 (54.87-107.60) |
| High-income North America | 0.93 (0.46-1.54) | 15.96 (7.84-26.04) | 4.92 (2.23-8.54) | 20.88 (10.16-33.89) |
| **Oceania** |  |  |  |  |
| Oceania | 8.39 (2.77-18.68) | 176.88 (57.68-400.22) | 10.82 (3.80-23.93) | 187.69 (61.88-427.46) |
| Australasia | 1.16 (0.67-1.75) | 16.84 (9.84-25.53) | 5.29 (2.88-8.26) | 22.13 (13.11-33.10) |

UI: uncertainty intervals; DALYs: Disability-Adjusted Life Years; YLDs: Years Lived with Disability; YLLs: Years of Life Lost.

**S6 Table.** Global Trends in Household Air Pollution-Induced Stroke Outcomes: EAPC Analysis Across 204 Countries for Deaths, DALYs, YLDs, and YLLs (1990-2021).

| Country | EAPC for Deaths (95% CI) | EAPC for DALYs (95% CI) | EAPC for YLDs (95% CI) | EAPC for YLLs (95% CI) |
| --- | --- | --- | --- | --- |
| China | 1.76 (1.24 to 2.27) | 1.67 (1.21 to 2.13) | 4.14 (3.74 to 4.53) | 1.49 (1.02 to 1.97) |
| Democratic People's Republic of Korea | -1.24 (-1.52 to -0.95) | -1.09 (-1.37 to -0.82) | -1.03 (-1.25 to -0.81) | -1.10 (-1.38 to -0.82) |
| Indonesia | 1.25 (1.11 to 1.40) | 0.86 (0.72 to 1.00) | 0.58 (0.41 to 0.76) | 0.88 (0.74 to 1.02) |
| Georgia | 1.05 (0.23 to 1.89) | 0.63 (-0.10 to 1.37) | 1.53 (0.53 to 2.54) | 0.57 (-0.15 to 1.30) |
| Taiwan (Province of China) | -5.24 (-5.52 to -4.96) | -4.57 (-4.82 to -4.31) | -1.74 (-2.10 to -1.38) | -5.18 (-5.44 to -4.92) |
| Cambodia | 0.58 (0.32 to 0.84) | 0.24 (-0.01 to 0.49) | 1.25 (1.03 to 1.47) | 0.19 (-0.06 to 0.44) |
| Lao People's Democratic Republic | 0.94 (0.64 to 1.23) | 0.68 (0.39 to 0.97) | 2.25 (2.01 to 2.50) | 0.60 (0.32 to 0.89) |
| Malaysia | -2.98 (-3.21 to -2.75) | -3.17 (-3.43 to -2.91) | -2.14 (-2.34 to -1.93) | -3.29 (-3.55 to -3.02) |
| Myanmar | 1.12 (0.86 to 1.38) | 0.88 (0.64 to 1.12) | 2.34 (2.07 to 2.61) | 0.81 (0.57 to 1.05) |
| Maldives | -4.83 (-5.21 to -4.44) | -5.37 (-5.75 to -5.00) | -3.02 (-3.43 to -2.61) | -5.59 (-5.97 to -5.21) |
| Philippines | -0.29 (-0.58 to 0.00) | 0.12 (-0.17 to 0.41) | 0.71 (0.49 to 0.93) | 0.07 (-0.23 to 0.38) |
| Thailand | -2.32 (-2.84 to -1.80) | -1.98 (-2.48 to -1.47) | -0.49 (-0.85 to -0.11) | -2.15 (-2.68 to -1.63) |
| Timor-Leste | 2.69 (2.31 to 3.06) | 2.55 (2.18 to 2.93) | 2.76 (2.40 to 3.12) | 2.54 (2.16 to 2.92) |
| Sri Lanka | 1.24 (0.85 to 1.64) | 0.89 (0.50 to 1.29) | 1.59 (1.33 to 1.85) | 0.83 (0.42 to 1.24) |
| Vietnam | 4.95 (4.60 to 5.29) | 4.81 (4.46 to 5.17) | 5.32 (5.05 to 5.60) | 4.79 (4.43 to 5.15) |
| Kiribati | 0.33(0.17 to 0.48) | 0.23(0.07 to 0.38) | 0.24(0.06 to 0.42) | 0.23(0.07 to 0.38) |
| Fiji | 0.51 (0.08 to 0.95) | 0.38 (0.00 to 0.77) | 1.02 (0.68 to 1.35) | 0.33 (-0.06 to 0.71) |
| Micronesia (Federated States of) | 0.23 (0.15 to 0.30) | 0.24 (0.16 to 0.31) | 0.86 (0.74 to 0.98) | 0.20 (0.13 to 0.27) |
| Marshall Islands | -0.39 (-0.60 to -0.19) | -0.36 (-0.58 to -0.15) | 0.02 (-0.19 to 0.24) | -0.39 (-0.60 to -0.17) |
| Papua New Guinea | -0.09 (-0.29 to 0.11) | -0.19 (-0.40 to 0.02) | 0.33 (0.12 to 0.54) | -0.21 (-0.42 to 0.00) |
| Samoa | 0.68 (0.57 to 0.78) | 0.76 (0.65 to 0.86) | 1.47 (1.31 to 1.62) | 0.70 (0.58 to 0.82) |
| Tonga | 0.98 (0.76 to 1.21) | 0.85 (0.64 to 1.06) | 1.13 (0.94 to 1.32) | 0.82 (0.60 to 1.03) |
| Solomon Islands | 0.20 (-0.01 to 0.40) | 0.24 (0.02 to 0.46) | 0.49 (0.33 to 0.64) | 0.22 (0.00 to 0.45) |
| Vanuatu | 1.28 (0.60 to 1.96) | 1.33 (0.64 to 2.03) | 2.20 (1.55 to 2.86) | 1.27 (0.58 to 1.97) |
| Azerbaijan | 1.52 (0.75 to 2.29) | 0.92 (0.24 to 1.60) | 1.73 (1.04 to 2.43) | 0.85 (0.17 to 1.54) |
| Armenia | -0.36 (-0.77 to 0.05) | -0.36 (-0.76 to 0.05) | 1.89 (1.34 to 2.45) | -0.61 (-1.02 to -0.20) |
| Kazakhstan | 0.17 (-0.23 to 0.58) | -0.29 (-0.66 to 0.08) | 0.18 (-0.12 to 0.48) | -0.33 (-0.72 to 0.07) |
| Kyrgyzstan | -1.85 (-2.34 to -1.35) | -1.71 (-2.14 to -1.28) | -0.36 (-1.24 to 0.52) | -1.80 (-2.21 to -1.39) |
| Mongolia | 3.47 (2.92 to 4.03) | 3.51 (2.96 to 4.06) | 5.52 (4.90 to 6.14) | 3.40 (2.84 to 3.95) |
| Tajikistan | 1.20 (0.47 to 1.93) | 0.90 (0.18 to 1.62) | 1.89 (0.93 to 2.86) | 0.83 (0.13 to 1.54) |
| Turkmenistan | 0.81 (0.31 to 1.32) | 0.92 (0.44 to 1.41) | 1.22 (0.82 to 1.61) | 0.90 (0.40 to 1.41) |
| Uzbekistan | 0.65 (0.32 to 0.99) | 0.38 (0.04 to 0.73) | 1.84 (1.42 to 2.26) | 0.25 (-0.10 to 0.61) |
| Albania | 1.11 (0.69 to 1.54) | 0.60 (0.19 to 1.01) | 1.29 (0.82 to 1.77) | 0.56 (0.15 to 0.97) |
| Bosnia and Herzegovina | 2.36 (1.67 to 3.06) | 2.14 (1.41 to 2.88) | 3.71 (2.83 to 4.61) | 1.99 (1.27 to 2.72) |
| Bulgaria | -2.72 (-3.04 to -2.39) | -2.91 (-3.21 to -2.61) | -1.49 (-1.70 to -1.28) | -2.99 (-3.30 to -2.68) |
| Croatia | -4.90 (-5.29 to -4.51) | -4.97 (-5.35 to -4.59) | -1.96 (-2.23 to -1.68) | -5.25 (-5.65 to -4.84) |
| Czechia | -7.07 (-7.57 to -6.57) | -6.64 (-7.02 to -6.26) | -2.68 (-2.97 to -2.39) | -7.17 (-7.60 to -6.75) |
| Hungary | -5.81 (-6.03 to -5.59) | -5.70 (-5.91 to -5.50) | -3.41 (-3.61 to -3.20) | -5.96 (-6.18 to -5.74) |
| North Macedonia | -0.79 (-1.33 to -0.24) | -1.38 (-1.81 to -0.95) | -1.19 (-1.33 to -1.05) | -1.39 (-1.84 to -0.94) |
| Montenegro | -0.30 (-0.51 to -0.10) | -1.01 (-1.18 to -0.83) | -1.31 (-1.52 to -1.09) | -0.99 (-1.17 to -0.81) |
| Poland | -4.78 (-5.10 to -4.45) | -4.63 (-4.95 to -4.31) | -1.71 (-1.93 to -1.49) | -4.87 (-5.20 to -4.53) |
| Romania | -3.41 (-3.85 to -2.96) | -3.49 (-3.94 to -3.03) | -1.60 (-1.88 to -1.32) | -3.62 (-4.08 to -3.14) |
| Serbia | -3.04 (-3.30 to -2.78) | -3.03 (-3.33 to -2.73) | -1.37 (-1.63 to -1.12) | -3.12 (-3.43 to -2.81) |
| Slovakia | -4.08 (-4.31 to -3.85) | -4.11 (-4.36 to -3.86) | -2.52 (-2.85 to -2.20) | -4.35 (-4.60 to -4.10) |
| Slovenia | -5.72 (-5.92 to -5.53) | -6.10 (-6.28 to -5.91) | -3.05 (-3.22 to -2.88) | -6.49 (-6.69 to -6.28) |
| Belarus | -4.13 (-4.59 to -3.67) | -4.26 (-4.77 to -3.75) | -2.94 (-3.18 to -2.70) | -4.39 (-4.93 to -3.85) |
| Estonia | -10.72 (-11.43 to -10.00) | -10.33 (-10.98 to -9.66) | -5.78 (-6.11 to -5.46) | -10.86 (-11.58 to -10.14) |
| Ukraine | -5.02 (-5.29 to -4.74) | -4.60 (-4.87 to -4.34) | -2.87 (-2.99 to -2.76) | -4.75 (-5.03 to -4.47) |
| Latvia | -5.27 (-5.54 to -5.00) | -5.43 (-5.73 to -5.13) | -2.93 (-3.06 to -2.80) | -5.63 (-5.95 to -5.30) |
| Lithuania | -4.82 (-5.24 to -4.39) | -4.81 (-5.24 to -4.38) | -1.94 (-2.65 to -1.23) | -5.21 (-5.64 to -4.78) |
| Republic of Moldova | -2.32 (-2.96 to -1.66) | -2.07 (-2.62 to -1.52) | 0.09 (-0.56 to 0.73) | -2.22 (-2.76 to -1.67) |
| Russian Federation | -5.69 (-6.25 to -5.13) | -5.51 (-6.08 to -4.94) | -3.47 (-3.62 to -3.33) | -5.63 (-6.22 to -5.03) |
| Brunei Darussalam | -2.23 (-3.05 to -1.40) | -2.56 (-3.36 to -1.76) | -2.08 (-2.72 to -1.44) | -2.63 (-3.46 to -1.80) |
| Japan | -3.99 (-4.37 to -3.62) | -3.18 (-3.54 to -2.82) | -0.90 (-1.23 to -0.57) | -3.74 (-4.09 to -3.39) |
| Republic of Korea | -6.05 (-6.40 to -5.70) | -5.84 (-6.20 to -5.48) | -2.61 (-2.94 to -2.28) | -6.42 (-6.77 to -6.07) |
| Singapore | -7.47 (-7.84 to -7.10) | -6.84 (-7.30 to -6.38) | -4.15 (-4.75 to -3.54) | -7.70 (-8.10 to -7.30) |
| New Zealand | -3.00 (-3.36 to -2.64) | -3.06 (-3.45 to -2.67) | -1.11 (-1.47 to -0.74) | -3.38 (-3.77 to -2.99) |
| Australia | -3.00 (-3.55 to -2.44) | -2.67 (-3.26 to -2.07) | -0.28 (-0.80 to 0.24) | -3.19 (-3.78 to -2.60) |
| Andorra | -4.67 (-5.03 to -4.31) | -4.71 (-5.05 to -4.36) | -3.63 (-4.03 to -3.23) | -4.93 (-5.28 to -4.57) |
| Belgium | -6.24 (-6.49 to -5.99) | -5.95 (-6.18 to -5.72) | -3.42 (-3.63 to -3.20) | -6.32 (-6.57 to -6.06) |
| Italy | -5.66 (-5.81 to -5.50) | -5.68 (-5.81 to -5.54) | -3.46 (-3.67 to -3.25) | -5.95 (-6.09 to -5.80) |
| Cyprus | -5.60 (-5.83 to -5.36) | -5.58 (-5.79 to -5.38) | -3.23 (-3.56 to -2.89) | -5.75 (-5.96 to -5.55) |
| Denmark | -6.03 (-6.43 to -5.63) | -6.11 (-6.51 to -5.71) | -4.38 (-4.68 to -4.08) | -6.39 (-6.81 to -5.96) |
| Finland | -7.56 (-7.95 to -7.16) | -7.42 (-7.83 to -7.01) | -5.20 (-5.80 to -4.60) | -7.92 (-8.31 to -7.53) |
| France | -6.00 (-6.24 to -5.76) | -5.53 (-5.78 to -5.28) | -2.74 (-3.05 to -2.42) | -6.04 (-6.30 to -5.78) |
| Greece | -5.97 (-6.44 to -5.49) | -5.53 (-5.92 to -5.13) | -2.81 (-3.15 to -2.47) | -5.81 (-6.22 to -5.39) |
| Germany | -6.71 (-6.90 to -6.53) | -6.15 (-6.33 to -5.97) | -3.51 (-3.82 to -3.21) | -6.73 (-6.90 to -6.56) |
| Sweden | -7.40 (-7.94 to -6.86) | -7.23 (-7.71 to -6.75) | -5.09 (-5.44 to -4.74) | -7.70 (-8.21 to -7.18) |
| Norway | -8.11 (-8.48 to -7.74) | -7.84 (-8.17 to -7.52) | -5.43 (-5.67 to -5.19) | -8.41 (-8.78 to -8.04) |
| Iceland | -6.38 (-6.89 to -5.87) | -6.31 (-6.74 to -5.87) | -4.44 (-4.82 to -4.06) | -6.75 (-7.21 to -6.29) |
| Ireland | -7.15 (-7.39 to -6.92) | -7.22 (-7.48 to -6.97) | -5.27 (-5.59 to -4.95) | -7.52 (-7.78 to -7.25) |
| Israel | -4.91 (-5.19 to -4.63) | -4.64 (-4.88 to -4.39) | -2.06 (-2.29 to -1.83) | -5.18 (-5.45 to -4.91) |
| Luxembourg | -7.90 (-8.27 to -7.54) | -7.88 (-8.22 to -7.54) | -5.17 (-5.49 to -4.85) | -8.18 (-8.54 to -7.82) |
| Netherlands | -5.64 (-5.92 to -5.36) | -5.77 (-6.07 to -5.46) | -4.42 (-4.72 to -4.13) | -6.03 (-6.35 to -5.71) |
| Portugal | -8.48 (-8.97 to -8.00) | -8.27 (-8.75 to -7.79) | -5.77 (-6.34 to -5.21) | -8.46 (-8.94 to -7.98) |
| Spain | -6.81 (-7.10 to -6.52) | -6.30 (-6.58 to -6.03) | -3.11 (-3.45 to -2.77) | -6.83 (-7.12 to -6.54) |
| Malta | -5.67 (-6.15 to -5.18) | -5.57 (-5.98 to -5.16) | -3.22 (-3.44 to -2.99) | -5.89 (-6.34 to -5.44) |
| Switzerland | -6.79 (-6.99 to -6.59) | -6.54 (-6.74 to -6.34) | -3.72 (-3.97 to -3.46) | -7.10 (-7.31 to -6.89) |
| United States | -5.32 (-5.78 to -4.86) | -5.12 (-5.56 to -4.67) | -4.15 (-4.70 to -3.59) | -5.37 (-5.80 to -4.94) |
| United Kingdom | -6.78 (-7.13 to -6.44) | -6.59 (-6.89 to -6.28) | -4.22 (-4.44 to -3.99) | -6.97 (-7.30 to -6.63) |
| Argentina | -4.32 (-4.65 to -3.99) | -4.37 (-4.69 to -4.05) | -2.52 (-2.76 to -2.28) | -4.59 (-4.92 to -4.26) |
| Chile | -2.06 (-2.34 to -1.78) | -2.19 (-2.45 to -1.92) | -0.35 (-0.67 to -0.02) | -2.43 (-2.70 to -2.16) |
| Uruguay | -4.30 (-4.62 to -3.97) | -4.44 (-4.77 to -4.11) | -3.27 (-3.57 to -2.97) | -4.58 (-4.92 to -4.24) |
| Canada | -6.49 (-6.93 to -6.04) | -5.90 (-6.29 to -5.52) | -3.79 (-4.18 to -3.40) | -6.58 (-6.98 to -6.17) |
| Belize | -0.82 (-1.39 to -0.24) | -0.95 (-1.49 to -0.41) | 0.16 (-0.02 to 0.35) | -1.01 (-1.56 to -0.45) |
| Bahamas | -1.66 (-1.82 to -1.49) | -1.74 (-1.88 to -1.60) | -0.57 (-0.71 to -0.43) | -1.80 (-1.95 to -1.66) |
| Antigua and Barbuda | -1.81 (-2.15 to -1.46) | -2.03 (-2.35 to -1.70) | -0.58 (-0.76 to -0.41) | -2.09 (-2.43 to -1.75) |
| Dominica | -0.35 (-0.56 to -0.15) | -0.37 (-0.60 to -0.14) | 0.10 (-0.06 to 0.25) | -0.39 (-0.62 to -0.16) |
| Cuba | -1.10 (-1.29 to -0.91) | -1.19 (-1.41 to -0.97) | -0.29 (-0.46 to -0.11) | -1.25 (-1.47 to -1.02) |
| Barbados | -1.65 (-2.00 to -1.31) | -1.62 (-1.95 to -1.29) | -0.41 (-0.62 to -0.20) | -1.68 (-2.01 to -1.34) |
| Grenada | -1.28 (-1.43 to -1.14) | -1.59 (-1.71 to -1.47) | -0.22 (-0.38 to -0.05) | -1.64 (-1.76 to -1.52) |
| Haiti | -0.59 (-0.75 to -0.42) | -0.71 (-0.89 to -0.53) | -0.18 (-0.34 to -0.03) | -0.72 (-0.90 to -0.54) |
| Saint Lucia | -2.95 (-3.39 to -2.50) | -2.73 (-3.10 to -2.35) | -0.62 (-0.79 to -0.46) | -2.82 (-3.21 to -2.44) |
| Dominican Republic | 1.12 (0.85 to 1.39) | 1.21 (0.97 to 1.44) | 1.68 (1.48 to 1.88) | 1.18 (0.94 to 1.42) |
| Jamaica | 0.17 (-0.24 to 0.58) | 0.04 (-0.38 to 0.47) | 0.51 (0.39 to 0.63) | 0.02 (-0.42 to 0.47) |
| Guyana | -0.28 (-0.43 to -0.13) | -0.54 (-0.68 to -0.39) | 0.33 (0.10 to 0.57) | -0.56 (-0.71 to -0.42) |
| Saint Vincent and the Grenadines | -0.95 (-1.17 to -0.74) | -1.03 (-1.22 to -0.83) | -0.29 (-0.45 to -0.14) | -1.06 (-1.26 to -0.85) |
| Trinidad and Tobago | -2.19 (-2.57 to -1.81) | -2.19 (-2.56 to -1.81) | -0.77 (-0.96 to -0.59) | -2.26 (-2.64 to -1.87) |
| Bolivia (Plurinational State of) | -3.45 (-3.72 to -3.18) | -3.76 (-4.04 to -3.47) | -2.54 (-2.88 to -2.21) | -3.81 (-4.09 to -3.52) |
| Suriname | -0.86 (-1.18 to -0.54) | -0.94 (-1.27 to -0.60) | -0.26 (-0.48 to -0.04) | -0.96 (-1.30 to -0.62) |
| Peru | -3.72 (-4.14 to -3.30) | -3.60 (-3.98 to -3.22) | -1.74 (-2.03 to -1.45) | -3.76 (-4.15 to -3.36) |
| Colombia | -5.07 (-5.27 to -4.87) | -4.98 (-5.17 to -4.79) | -2.91 (-3.14 to -2.69) | -5.17 (-5.36 to -4.97) |
| Ecuador | -4.49 (-5.07 to -3.92) | -4.78 (-5.35 to -4.21) | -3.40 (-3.90 to -2.88) | -4.89 (-5.46 to -4.31) |
| El Salvador | -1.64 (-1.92 to -1.35) | -1.78 (-2.06 to -1.50) | -0.38 (-0.70 to -0.06) | -1.89 (-2.19 to -1.59) |
| Honduras | 0.84 (0.49 to 1.20) | 0.41 (0.11 to 0.72) | -0.49 (-0.68 to -0.30) | 0.44 (0.13 to 0.75) |
| Costa Rica | -3.32 (-3.63 to -3.01) | -3.22 (-3.51 to -2.93) | -1.78 (-2.11 to -1.44) | -3.39 (-3.69 to -3.09) |
| Nicaragua | -1.17 (-1.50 to -0.84) | -1.25 (-1.54 to -0.96) | -0.20 (-0.40 to 0.00) | -1.34 (-1.64 to -1.03) |
| Guatemala | -2.57 (-3.02 to -2.13) | -2.75 (-3.18 to -2.31) | -1.34 (-1.54 to -1.13) | -2.83 (-3.27 to -2.38) |
| Mexico | -4.71 (-4.98 to -4.45) | -4.39 (-4.63 to -4.14) | -3.35 (-3.60 to -3.09) | -4.49 (-4.74 to -4.24) |
| Panama | -3.09 (-3.50 to -2.69) | -3.09 (-3.43 to -2.75) | -1.96 (-2.32 to -1.60) | -3.18 (-3.52 to -2.83) |
| Venezuela (Bolivarian Republic of) | -2.72 (-2.90 to -2.54) | -2.89 (-3.08 to -2.69) | -2.08 (-2.36 to -1.80) | -2.94 (-3.14 to -2.74) |
| Bahrain | -2.83 (-3.35 to -2.30) | -3.16 (-3.62 to -2.69) | -0.98 (-1.11 to -0.84) | -3.28 (-3.76 to -2.79) |
| Paraguay | -1.36 (-1.51 to -1.21) | -1.54 (-1.70 to -1.39) | -0.87 (-1.04 to -0.69) | -1.58 (-1.73 to -1.42) |
| Iran (Islamic Republic of) | -1.97 (-2.10 to -1.84) | -1.98 (-2.10 to -1.86) | -0.17 (-0.32 to -0.01) | -2.13 (-2.26 to -2.01) |
| Brazil | -3.67 (-3.89 to -3.44) | -3.84 (-4.07 to -3.62) | -1.98 (-2.22 to -1.74) | -3.94 (-4.16 to -3.71) |
| Algeria | -1.05 (-1.24 to -0.85) | -1.39 (-1.60 to -1.17) | -0.26 (-0.50 to -0.03) | -1.48 (-1.69 to -1.26) |
| Jordan | -2.05 (-2.40 to -1.69) | -2.16 (-2.51 to -1.81) | 0.89 (0.61 to 1.17) | -2.48 (-2.86 to -2.11) |
| Egypt | 0.02 (-0.28 to 0.31) | -0.09 (-0.37 to 0.18) | 1.34 (1.11 to 1.58) | -0.15 (-0.43 to 0.13) |
| Lebanon | -3.15 (-3.42 to -2.88) | -3.14 (-3.45 to -2.83) | 0.81 (0.61 to 1.01) | -3.56 (-3.88 to -3.24) |
| Iraq | -0.46 (-0.65 to -0.27) | -0.84 (-1.02 to -0.66) | 0.43 (0.15 to 0.70) | -0.90 (-1.08 to -0.72) |
| Morocco | 0.91 (0.50 to 1.31) | 0.65 (0.24 to 1.06) | 1.15 (0.71 to 1.58) | 0.62 (0.21 to 1.03) |
| Kuwait | -0.84 (-2.00 to 0.32) | -0.98 (-1.98 to 0.04) | -0.02 (-0.31 to 0.26) | -1.17 (-2.31 to -0.03) |
| Palestine | -0.66 (-1.16 to -0.15) | -0.78 (-1.24 to -0.32) | 0.63 (0.43 to 0.83) | -0.85 (-1.32 to -0.37) |
| Qatar | -4.08 (-4.85 to -3.31) | -3.93 (-4.57 to -3.27) | -1.71 (-1.93 to -1.49) | -4.18 (-4.88 to -3.47) |
| Saudi Arabia | -1.11 (-1.32 to -0.89) | -0.98 (-1.20 to -0.76) | 0.46 (0.31 to 0.61) | -1.05 (-1.28 to -0.83) |
| Libya | 0.64 (0.25 to 1.03) | 0.49 (0.15 to 0.84) | 0.42 (0.06 to 0.79) | 0.50 (0.15 to 0.85) |
| Yemen | 0.56 (0.39 to 0.73) | 0.30 (0.13 to 0.46) | 1.41 (1.15 to 1.68) | 0.26 (0.10 to 0.42) |
| Turkey | -1.51 (-1.94 to -1.08) | -2.05 (-2.39 to -1.71) | -0.78 (-0.90 to -0.66) | -2.17 (-2.54 to -1.80) |
| Syrian Arab Republic | -0.96 (-1.18 to -0.75) | -1.27 (-1.51 to -1.04) | -0.42 (-0.63 to -0.22) | -1.33 (-1.57 to -1.08) |
| Oman | -1.12 (-1.42 to -0.81) | -1.59 (-1.79 to -1.38) | -0.08 (-0.24 to 0.08) | -1.71 (-1.92 to -1.49) |
| United Arab Emirates | 0.46 (-0.23 to 1.16) | -0.45 (-1.00 to 0.10) | -0.71 (-0.83 to -0.58) | -0.44 (-1.03 to 0.16) |
| India | 2.11 (1.69 to 2.53) | 1.96 (1.57 to 2.34) | 2.80 (2.44 to 3.15) | 1.91 (1.52 to 2.30) |
| Tunisia | -0.92 (-1.30 to -0.54) | -0.93 (-1.30 to -0.55) | 0.60 (0.24 to 0.97) | -1.03 (-1.41 to -0.66) |
| Bangladesh | 0.39 (0.04 to 0.74) | 0.12 (-0.22 to 0.46) | 1.11 (0.61 to 1.62) | 0.08 (-0.26 to 0.41) |
| Nepal | 0.46 (-0.10 to 1.03) | 0.25 (-0.31 to 0.81) | 1.06 (0.66 to 1.47) | 0.21 (-0.36 to 0.78) |
| Afghanistan | -1.14 (-1.55 to -0.72) | -1.30 (-1.70 to -0.89) | -0.37 (-0.91 to 0.18) | -1.33 (-1.73 to -0.92) |
| Congo | 0.84 (0.61 to 1.06) | 0.63 (0.40 to 0.86) | 1.64 (1.40 to 1.88) | 0.56 (0.33 to 0.80) |
| Central African Republic | -0.50 (-0.64 to -0.35) | -0.59 (-0.73 to -0.44) | -0.33 (-0.45 to -0.20) | -0.60 (-0.74 to -0.45) |
| Pakistan | 1.21 (0.88 to 1.54) | 1.19 (0.86 to 1.53) | 1.57 (1.26 to 1.88) | 1.16 (0.83 to 1.51) |
| Angola | 2.17 (1.88 to 2.46) | 1.97 (1.68 to 2.27) | 3.09 (2.74 to 3.45) | 1.90 (1.61 to 2.19) |
| Democratic Republic of the Congo | -0.60 (-0.82 to -0.38) | -0.65 (-0.87 to -0.43) | -0.59 (-0.84 to -0.34) | -0.65 (-0.87 to -0.44) |
| Bhutan | 3.05 (2.69 to 3.42) | 2.74 (2.38 to 3.10) | 3.91 (3.53 to 4.29) | 2.66 (2.30 to 3.02) |
| Equatorial Guinea | 2.57 (1.86 to 3.28) | 2.21 (1.53 to 2.89) | 4.72 (3.62 to 5.82) | 2.04 (1.38 to 2.69) |
| Eritrea | -0.94 (-1.21 to -0.66) | -1.21 (-1.47 to -0.95) | -0.08 (-0.44 to 0.28) | -1.26 (-1.52 to -1.00) |
| Burundi | -3.02 (-3.38 to -2.66) | -3.21 (-3.59 to -2.82) | -1.76 (-1.89 to -1.64) | -3.28 (-3.67 to -2.88) |
| Djibouti | 0.38 (0.18 to 0.57) | 0.27 (0.06 to 0.47) | 1.15 (0.95 to 1.36) | 0.20 (0.00 to 0.41) |
| Comoros | -1.57 (-1.75 to -1.39) | -1.75 (-1.95 to -1.55) | -0.50 (-0.67 to -0.33) | -1.84 (-2.06 to -1.63) |
| Kenya | 1.90 (1.59 to 2.22) | 1.91 (1.57 to 2.24) | 1.56 (1.26 to 1.86) | 1.94 (1.60 to 2.28) |
| Ethiopia | -0.46 (-0.63 to -0.30) | -0.84 (-1.03 to -0.65) | 1.07 (0.84 to 1.29) | -0.95 (-1.13 to -0.76) |
| Gabon | -0.75 (-0.90 to -0.60) | -0.90 (-1.05 to -0.75) | -0.28 (-0.40 to -0.17) | -0.95 (-1.10 to -0.80) |
| Rwanda | -3.80 (-4.18 to -3.42) | -4.21 (-4.62 to -3.80) | -1.59 (-1.73 to -1.45) | -4.34 (-4.76 to -3.91) |
| South Africa | 0.80 (0.35 to 1.25) | 0.37 (-0.03 to 0.77) | -0.48 (-0.58 to -0.38) | 0.45 (0.02 to 0.89) |
| Mauritius | -4.48 (-5.12 to -3.82) | -4.25 (-4.86 to -3.62) | -1.82 (-2.22 to -1.43) | -4.50 (-5.16 to -3.83) |
| Madagascar | 1.21 (0.89 to 1.52) | 1.14 (0.82 to 1.46) | 1.71 (1.37 to 2.05) | 1.11 (0.79 to 1.43) |
| Malawi | 0.28 (0.00 to 0.56) | 0.22 (-0.08 to 0.52) | 0.37 (0.20 to 0.55) | 0.21 (-0.10 to 0.52) |
| Seychelles | -1.73 (-2.01 to -1.44) | -1.97 (-2.25 to -1.68) | -1.24 (-1.59 to -0.90) | -2.04 (-2.32 to -1.76) |
| Somalia | -0.19 (-0.62 to 0.23) | -0.28 (-0.70 to 0.15) | 0.44 (0.01 to 0.89) | -0.31 (-0.73 to 0.12) |
| United Republic of Tanzania | 1.78 (1.55 to 2.01) | 1.45 (1.25 to 1.65) | 2.95 (2.70 to 3.21) | 1.34 (1.15 to 1.54) |
| Zambia | 0.56 (-0.30 to 1.43) | 0.44 (-0.40 to 1.29) | 1.30 (0.42 to 2.18) | 0.40 (-0.44 to 1.24) |
| Uganda | -0.71 (-0.99 to -0.42) | -0.76 (-1.07 to -0.46) | 1.02 (0.84 to 1.20) | -0.89 (-1.20 to -0.57) |
| Mozambique | 1.80 (1.51 to 2.10) | 1.96 (1.65 to 2.28) | 1.39 (1.25 to 1.53) | 1.99 (1.66 to 2.31) |
| Lesotho | 2.94 (2.57 to 3.31) | 3.05 (2.67 to 3.43) | 1.53 (1.28 to 1.78) | 3.11 (2.72 to 3.51) |
| Botswana | -0.40 (-0.89 to 0.10) | -0.56 (-1.05 to -0.06) | 1.22 (0.87 to 1.57) | -0.71 (-1.22 to -0.19) |
| Namibia | 0.91 (0.67 to 1.15) | 0.73 (0.48 to 0.98) | 1.24 (1.04 to 1.43) | 0.70 (0.44 to 0.96) |
| Eswatini | 0.87 (0.38 to 1.37) | 0.88 (0.33 to 1.43) | 0.66 (0.47 to 0.85) | 0.89 (0.31 to 1.46) |
| Cabo Verde | 3.42 (2.78 to 4.07) | 3.02 (2.44 to 3.61) | 3.31 (2.83 to 3.79) | 2.99 (2.39 to 3.60) |
| Benin | -0.31 (-0.72 to 0.10) | -0.43 (-0.85 to -0.01) | -0.04 (-0.39 to 0.31) | -0.45 (-0.88 to -0.02) |
| Zimbabwe | 0.46 (-0.07 to 0.98) | 0.60 (0.05 to 1.16) | -0.95 (-1.20 to -0.71) | 0.70 (0.12 to 1.28) |
| Chad | 1.28 (0.99 to 1.57) | 1.21 (0.92 to 1.51) | 0.96 (0.71 to 1.20) | 1.23 (0.93 to 1.53) |
| Cameroon | 0.45 (0.18 to 0.73) | 0.37 (0.09 to 0.66) | 0.53 (0.22 to 0.84) | 0.37 (0.08 to 0.65) |
| Ghana | 2.03 (1.70 to 2.36) | 1.87 (1.53 to 2.20) | 1.98 (1.54 to 2.42) | 1.86 (1.53 to 2.19) |
| Burkina Faso | 0.78 (0.48 to 1.08) | 0.63 (0.34 to 0.93) | 0.60 (0.35 to 0.84) | 0.64 (0.34 to 0.93) |
| Liberia | 0.36 (-0.02 to 0.74) | 0.29 (-0.10 to 0.67) | 0.25 (-0.16 to 0.66) | 0.29 (-0.09 to 0.67) |
| Côte d'Ivoire | 0.13 (-0.24 to 0.50) | -0.01 (-0.40 to 0.38) | 0.10 (-0.31 to 0.50) | -0.02 (-0.40 to 0.37) |
| Guinea | 0.36 (0.14 to 0.59) | 0.30 (0.07 to 0.52) | 0.05 (-0.16 to 0.27) | 0.31 (0.09 to 0.53) |
| Gambia | -0.10 (-0.28 to 0.08) | -0.30 (-0.49 to -0.11) | -0.53 (-0.69 to -0.37) | -0.28 (-0.48 to -0.09) |
| Guinea-Bissau | -0.55 (-0.72 to -0.39) | -0.76 (-0.91 to -0.60) | -0.65 (-0.81 to -0.49) | -0.76 (-0.92 to -0.60) |
| Mali | 0.49 (0.23 to 0.76) | 0.34 (0.08 to 0.60) | 0.46 (0.26 to 0.65) | 0.33 (0.07 to 0.60) |
| Niger | -0.13 (-0.37 to 0.11) | -0.33 (-0.57 to -0.09) | -0.57 (-0.77 to -0.36) | -0.32 (-0.56 to -0.07) |
| Mauritania | -0.16 (-0.43 to 0.10) | -0.43 (-0.69 to -0.17) | 0.19 (0.02 to 0.37) | -0.47 (-0.74 to -0.20) |
| Nigeria | -0.14 (-0.65 to 0.38) | -0.34 (-0.83 to 0.15) | 1.42 (0.89 to 1.96) | -0.46 (-0.95 to 0.03) |
| Togo | 0.01 (-0.36 to 0.38) | -0.06 (-0.43 to 0.31) | -0.30 (-0.72 to 0.12) | -0.05 (-0.42 to 0.32) |
| Senegal | -2.44 (-3.20 to -1.68) | -2.63 (-3.38 to -1.87) | -2.53 (-3.25 to -1.80) | -2.63 (-3.39 to -1.88) |
| American Samoa | -1.03 (-1.33 to -0.73) | -1.00 (-1.30 to -0.70) | -0.62 (-0.87 to -0.37) | -1.04 (-1.35 to -0.73) |
| Sierra Leone | 0.17 (-0.14 to 0.47) | 0.14 (-0.17 to 0.46) | 0.02 (-0.33 to 0.36) | 0.15 (-0.16 to 0.47) |
| Sao Tome and Principe | 2.83 (2.51 to 3.16) | 2.61 (2.31 to 2.92) | 2.63 (2.29 to 2.97) | 2.61 (2.31 to 2.92) |
| Bermuda | -5.28 (-5.70 to -4.86) | -5.17 (-5.57 to -4.77) | -3.09 (-3.49 to -2.68) | -5.38 (-5.78 to -4.98) |
| Greenland | -4.74 (-5.20 to -4.27) | -4.67 (-5.11 to -4.23) | -3.49 (-3.84 to -3.14) | -4.80 (-5.24 to -4.35) |
| Monaco | -3.19 (-3.98 to -2.40) | -3.06 (-3.87 to -2.24) | -1.46 (-2.40 to -0.50) | -3.26 (-4.06 to -2.45) |
| Cook Islands | -3.60 (-4.07 to -3.12) | -3.37 (-3.86 to -2.88) | -1.42 (-1.78 to -1.07) | -3.63 (-4.13 to -3.13) |
| Guam | -1.93 (-2.82 to -1.02) | -0.76 (-1.55 to 0.04) | 0.69 (0.16 to 1.22) | -1.04 (-1.88 to -0.18) |
| Niue | -2.96 (-3.35 to -2.57) | -3.02 (-3.42 to -2.62) | -2.29 (-2.64 to -1.95) | -3.09 (-3.50 to -2.69) |
| Palau | -0.34 (-1.22 to 0.55) | -0.40 (-1.27 to 0.48) | 0.18 (-0.67 to 1.04) | -0.45 (-1.32 to 0.43) |
| Saint Kitts and Nevis | -3.45 (-3.88 to -3.02) | -3.75 (-4.22 to -3.27) | -2.93 (-3.28 to -2.58) | -3.78 (-4.25 to -3.30) |
| Nauru | -1.30 (-2.06 to -0.54) | -1.29 (-2.08 to -0.50) | -1.07 (-1.55 to -0.59) | -1.31 (-2.11 to -0.50) |
| Northern Mariana Islands | -1.38 (-2.10 to -0.66) | -1.36 (-2.06 to -0.65) | -0.38 (-0.91 to 0.15) | -1.46 (-2.18 to -0.73) |
| Puerto Rico | -4.13 (-4.58 to -3.68) | -3.59 (-4.05 to -3.12) | -0.91 (-1.30 to -0.52) | -3.91 (-4.38 to -3.43) |
| Tokelau | -3.04 (-3.40 to -2.68) | -2.98 (-3.35 to -2.62) | -1.70 (-2.05 to -1.36) | -3.08 (-3.44 to -2.71) |
| San Marino | -5.07 (-5.63 to -4.50) | -4.69 (-5.18 to -4.19) | -2.97 (-3.32 to -2.63) | -5.02 (-5.55 to -4.49) |
| Tuvalu | 0.07 (-0.29 to 0.42) | 0.05 (-0.30 to 0.40) | 1.16 (0.76 to 1.57) | -0.01 (-0.36 to 0.33) |
| South Sudan | -2.39 (-2.74 to -2.04) | -2.46 (-2.78 to -2.14) | -1.84 (-2.45 to -1.23) | -2.50 (-2.80 to -2.19) |
| United States Virgin Islands | -2.84 (-3.21 to -2.47) | -2.76 (-3.15 to -2.37) | -0.59 (-0.89 to -0.30) | -2.91 (-3.30 to -2.51) |
| Sudan | 1.46 (1.23 to 1.69) | 1.32 (1.09 to 1.54) | 3.10 (2.82 to 3.38) | 1.23 (1.00 to 1.45) |

EAPC: Estimated Annual Percentage Change; CI: Confidence Interval; DALYs: Disability-Adjusted Life Years; YLDs: Years Lived with Disability; YLLs: Years of Life Lost.

**S7 Table.** Global Burden of Disease Metrics for 1990, 2021, and 2040 (Predicted)

| Measure | 1990 | | 2021 | | 2040 (Predicted) | |
| --- | --- | --- | --- | --- | --- | --- |
|  | Number of Cases | ASR/100000 | Number of Cases | ASR/100000 | Number of Cases | ASR/100000 |
| Deaths | 617,907 | 17.13 | 1,230,852 | 14.78 | 1,788,033 | 11.91 |
| YLDs | 925,788 | 21.93 | 2,425,770 | 28.22 | 3,285,345 | 24.40 |
| YLLs | 12,956,224 | 324.30 | 24,353,661 | 284.64 | 31,388,255 | 224.64 |
| DALYs | 13,882,012 | 346.22 | 26,779,431 | 312.85 | 34,758,511 | 249.77 |

ASR: Age-Standardized Rate; YLDs: Years Lived with Disability; YLLs: Years of Life Lost; DALYs: Disability-Adjusted Life Years.
