# Supplementary figures and images for: Global stroke burden attributable to household air pollution: Insights from GBD 2021 and projections to 2040
Source: PLoS One. 2025 Jul 29;20(7):e0327976. doi: 10.1371/journal.pone.0327976 (PMC12306745; doi:10.1371/journal.pone.0327976)

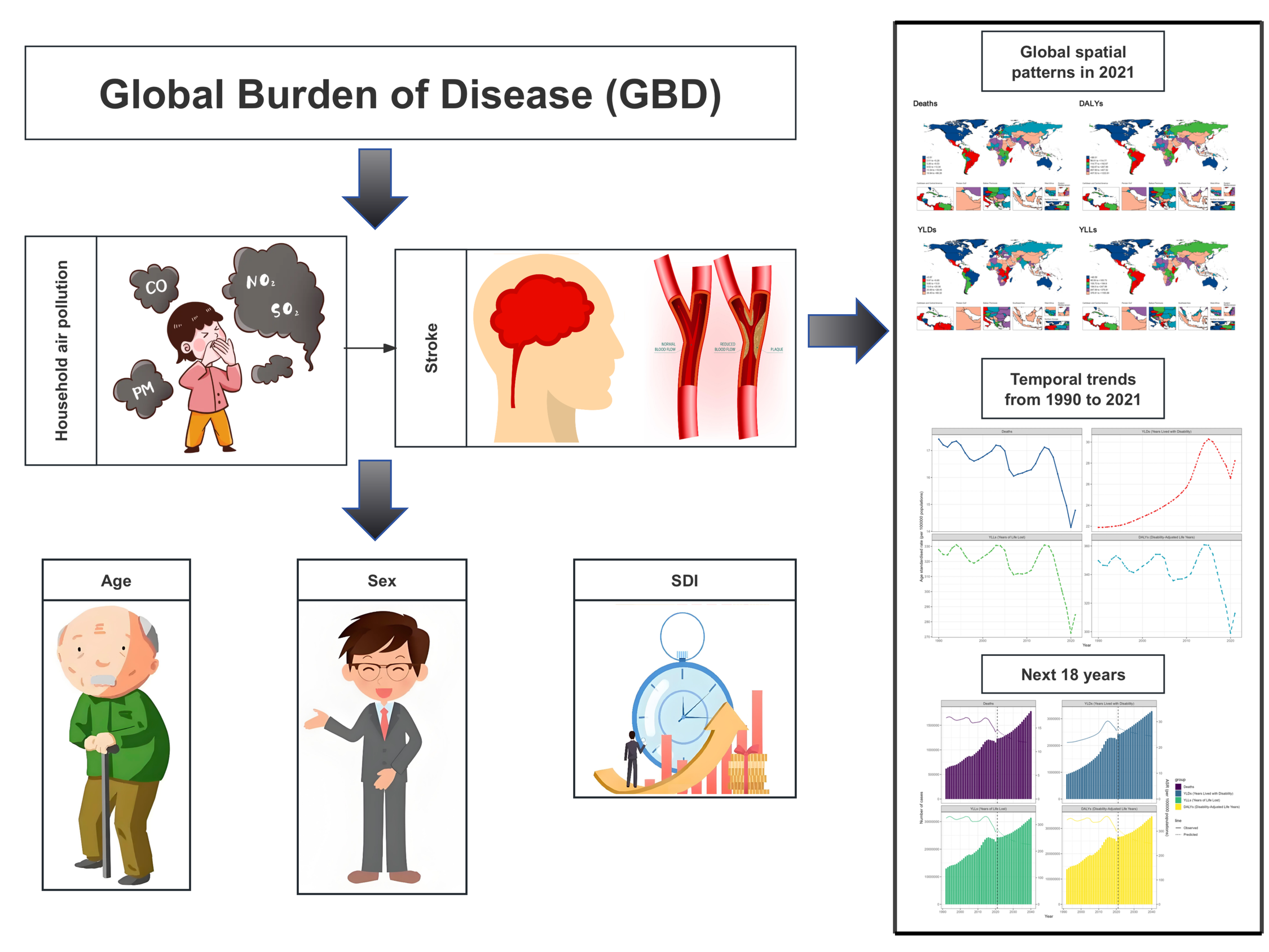

Supplement: S2 File — Global Burden of Disease (GBD) Analysis for Household Air Pollution (HAP)-Induced Stroke. This graphical abstract illustrates the relationship between HAP and stroke, based on GBD data. The left panel depicts key pollutants from HAP, such as particulate matter (PM), carbon monoxide (CO), nitrogen oxides (NOx), and sulfur dioxide (SO2), which contribute to stroke. The middle section shows the progression to stroke, while the lower section highlights the influence of factors such as age, sex, and socio-demographic index (SDI). The right panel presents global spatial patterns of stroke-related mortality, Disability-Adjusted Life Years (DALYs), Years Lived with Disability (YLDs), and Years of Life Lost (YLLs) in 2021, temporal trends from 1990 to 2021, and projections for the next 19 years. (TIF) [file pone.0327976.s002.tif]
