# Supplementary material for: Global stroke burden attributable to household air pollution: Insights from GBD 2021 and projections to 2040
Source: PLoS One. 2025 Jul 29;20(7):e0327976. doi: 10.1371/journal.pone.0327976 (PMC12306745; doi:10.1371/journal.pone.0327976)
Supplement: Attachment — Submitted filename: review HAP.docx [file pone.0327976.s003.docx]

The study provides a valuable update on the global stroke burden related to household air pollution (HAP), using the latest GBD 2021 data. However, while the projections to 2040 add a predictive aspect, it would be beneficial to clarify how this study substantially extends prior GBD-based research.

Abstract:

The conclusion states that “higher economic development and adoption of clean energy reduce HAP-related stroke burden.” However, since this study is observational and not an interventional trial, the phrasing should clarify that this can be a hypothesis rather than a direct causation.

The results mention “predictions indicate a gradual reduction in age-standardized rates.” Could the authors briefly state which statistical model was used for this projection in the abstract?

Method: It is mentioned that stroke burden below 25 years old was assumed negligible. Is there any literature supporting this cutoff, or was this assumption made based on data limitations?

results:

The findings suggest a global decline in age-standardized rates, yet absolute numbers continue to rise. Could this paradox be further elaborated upon? For example, how do population growth and aging contribute to the observed trends in the Discussion section?

all figures are too small and blurred, this makes interpretation very difficult.

Discussion:

The study finds that males experience a higher burden of HAP-induced stroke. However, it is unclear if this is due to increased exposure to air pollution or other co-existing risk factors (e.g., smoking, hypertension). Could this be discussed further?

Was occupational exposure considered as a potential confounder? In some developing regions, men may be more likely to work in high-exposure settings, which could affect results.

The study finds that middle-SDI regions have the highest burden. However, previous research often highlights low-SDI regions as suffering the most from air pollution exposure. Could the authors discuss why their findings diverge?

Some countries (e.g., Vietnam, Mongolia) experienced an increase in stroke burden due to HAP, while others (e.g., Estonia, Norway) showed a sharp decline. What policy or environmental factors might explain these differences?
