# Supplementary material for: Global stroke burden attributable to household air pollution: Insights from GBD 2021 and projections to 2040
Source: PLoS One. 2025 Jul 29;20(7):e0327976. doi: 10.1371/journal.pone.0327976 (PMC12306745; doi:10.1371/journal.pone.0327976)
Supplement: Attachment — Submitted filename: Response to Reviewers.docx [file pone.0327976.s005.docx]

**Response to Reviewers**

1. **Academic editor:**

**Reviewer point #1:** We note that Figure 7 in your submission contains map images which may be copyrighted. We require you to either (1) present written permission from the copyright holder to publish these figures specifically under the CC BY 4.0 license, or (2) remove the figures from your submission.

**Author response #1:** We thank the reviewer for raising this important concern regarding Figure 7 in our manuscript. We would like to clarify that Figure 7, which displays the global burden of stroke attributed to household air pollution across 204 countries and territories, was entirely generated by our research team using R statistical software (version 4.3.1). These maps were created using open-source R packages (e.g., ggplot2, sf, or maps) and publicly available GBD 2021 data from the Global Health Data Exchange GBD Results Tool (http://ghdx.healthdata.org/gbd-results-tool), as referenced in our methods section. No copyrighted map images were incorporated or adapted from external sources. Since these visualizations were produced as original work by the authors using open data sources and software tools, they are not subject to external copyright restrictions and can be published under the CC BY 4.0 license as part of our manuscript. We hope this clarification adequately addresses the reviewer's concern.

**Reviewer point #2:** The study provides a valuable update on the global stroke burden related to household air pollution (HAP), using the latest GBD 2021 data. However, while the projections to 2040 add a predictive aspect, it would be beneficial to clarify how this study substantially extends prior GBD-based research.

**Author response #2:** We sincerely thank the reviewer for recognizing the value of our study and for providing constructive feedback. We agree that clarifying how our study extends prior GBD-based research is essential to highlight its novelty and significance. To address this comment, we have revised the manuscript in the following ways:

1) Introduction (Revised Paragraph):

We have explicitly compared our work to prior GBD-based studies, notably Lu et al. (2021), which used GBD 2019 data to estimate 600,000 stroke deaths and 14.7 million DALYs in 2019. We now emphasize that our study leverages the updated GBD 2021 dataset, incorporates detailed gender and age stratifications, highlights the unique increasing trend in years lived with disability (YLDs), and extends projections to 2040 using the Nordpred model. These additions clarify how our study goes beyond previous analyses by providing a more comprehensive and forward-looking assessment (see revised Introduction, second paragraph).

2) Discussion (Revised Paragraph):

In the Discussion, we further elaborate on the extensions of our study compared to Lu et al. (2021). We note that while their work was foundational, it is now outdated and did not address the significant rise in YLDs (EAPC=1.11) or provide long-term projections. Our study reports a higher burden (1.23 million deaths and 26.78 million DALYs in 2021) based on GBD 2021 data, offers new insights into the disability burden through YLDs analysis, and extends the temporal scope to 2040. These advancements provide a more complete picture of the evolving HAP-related stroke burden and its implications for future public health strategies (see revised Discussion, second paragraph).

These revisions strengthen the manuscript by clearly delineating how our study builds upon and extends prior GBD-based research, offering both updated estimates and novel predictive insights. We are grateful for this suggestion and welcome any further feedback.

**Reviewer point #3:** Abstract: The conclusion states that “higher economic development and adoption of clean energy reduce HAP-related stroke burden.” However, since this study is observational and not an interventional trial, the phrasing should clarify that this can be a hypothesis rather than a direct causation.

**Author response #3:** We appreciate the reviewer's thoughtful comment regarding the conclusion statement in our abstract. The reviewer correctly points out that our study is observational and not an interventional trial, so we should be cautious about implying causation. We agree that our original statement, "Higher economic development and adoption of clean energy reduce HAP-related stroke burden," should be rephrased to reflect the associational nature of our findings better rather than suggesting direct causation.

We have revised the conclusion in the abstract to clarify that our observation suggests an association between economic development, clean energy adoption, and reduced HAP-related stroke burden, rather than making a direct causal claim. The revised text now appropriately reflects the observational nature of our study while still conveying the important relationships we observed in the data.

**Reviewer point #4:** Abstract: The results mention “predictions indicate a gradual reduction in age-standardized rates.” Could the authors briefly state which statistical model was used for this projection in the abstract?

**Author response #4:** We appreciate the reviewer's valuable feedback. We agree that the statistical model used for the projection should be mentioned in the abstract for clarity. We have added this information to the abstract as suggested. We used the Nordpred model for our projections, which is an improved version of the Age-Period-Cohort (APC) regression framework specifically designed for predicting trends in health outcomes. This information was previously included in our Methods section but was missing from the abstract. We have now added this detail to ensure methodological transparency in the abstract.

**Reviewer point #5:** Method: It is mentioned that stroke burden below 25 years old was assumed negligible. Is there any literature supporting this cutoff, or was this assumption made based on data limitations?

**Author response #5:** We thank the reviewer for this insightful question. We have revised our methodology section to provide better justification for our age cutoff. Our decision to use 25 years as the lower age limit is supported by both epidemiological evidence and previous research methodologies. Stroke incidence is known to increase significantly with age, with the vast majority of cases occurring in individuals over 25 years. This approach aligns with methodologies used in similar GBD-based analyses of stroke burden, including Hu et al. (2024), Lu et al. (2021) and Jiang et al. (2020), which employed similar age stratification approaches. We have updated the Methods section to provide this clarification and added appropriate references.

**Reviewer point #6:** Results: The findings suggest a global decline in age-standardized rates, yet absolute numbers continue to rise. Could this paradox be further elaborated upon? For example, how do population growth and aging contribute to the observed trends in the Discussion section?

**Author response #6:** We thank the reviewer for their valuable comment regarding the need to clarify the paradox between declining age-standardized rates and rising absolute numbers of stroke burden attributable to household air pollution (HAP). In response to this suggestion, we have significantly refined the Discussion section to provide a more comprehensive explanation of this observation. Initially, we expanded the third paragraph of the Discussion to highlight the roles of population growth and aging as key drivers of the increasing absolute burden, despite reductions in age-standardized rates. Building on the reviewer's feedback, we have further enriched this discussion by addressing how the rising absolute numbers may reflect the limited effectiveness of current primary stroke and cardiovascular disease prevention strategies, particularly in low- and middle-income countries (LMICs), as well as disparities in stroke care delivery, accessibility, and workforce capacity in these regions. These systemic factors, combined with demographic trends, provide a more holistic explanation for the observed patterns. The revised paragraph (paragraph 3 of the Discussion section) is presented below. We are grateful for the reviewer's suggestion, which has significantly improved the depth and clarity of our analysis.

**Reviewer point #7:** Results: All figures are too small and blurred, this makes interpretation very difficult.

**Author response #7:** We sincerely thank the reviewer for pointing out the issue regarding the size and clarity of the figures. We acknowledge that the figures in the initial submission were not sufficiently clear, which may have hindered their interpretation. To address this concern, we have revised all figures to ensure they are of higher resolution and appropriately sized for better readability. We have increased the resolution to 300 dpi and adjusted the dimensions to make the text, labels, and data points more legible. We hope these improvements will enhance the clarity and usability of the figures in the revised manuscript. Thank you again for your valuable feedback, which has helped us improve the quality of our work.

**Reviewer point #8:** Discussion: The study finds that males experience a higher burden of HAP-induced stroke. However, it is unclear if this is due to increased exposure to air pollution or other co-existing risk factors (e.g., smoking, hypertension). Could this be discussed further?

**Author response #8:** We appreciate the reviewer’s insightful comment. We have expanded the discussion on potential drivers of the gender disparity in the revised manuscript (Discussion section, paragraph 4). Specifically, we now elaborate on the interplay between HAP exposure, occupational risks, and behavioral factors. We highlight that while women may experience prolonged indoor exposure due to traditional cooking roles, men’s higher prevalence of smoking, hypertension, and occupational exposures (e.g., in high-pollution industries) likely contribute to their elevated stroke burden. Citations to supporting literature have been added to strengthen these points.

**Reviewer point #9:** Discussion: Was occupational exposure considered as a potential confounder? In some developing regions, men may be more likely to work in high-exposure settings, which could affect results.

**Author response #9:** We thank the reviewer’s thoughtful comments and suggestions that have helped improve our manuscript. While the GBD 2021 dataset does not explicitly adjust for occupational exposure, we acknowledge this limitation and have added a discussion on its potential confounding effects (Discussion section, paragraph 4). We propose that occupational exposure in high-risk industries (e.g., mining, construction) may synergize with HAP to exacerbate stroke risk in males. We emphasize the need for future studies to integrate occupational exposure data for a more comprehensive risk assessment.

**Reviewer point #10:** Discussion: The study finds that middle-SDI regions have the highest burden. However, previous research often highlights low-SDI regions as suffering the most from air pollution exposure. Could the authors discuss why their findings diverge?

**Author response #10:** We appreciate the reviewer’s request to explain why our finding—that middle-SDI regions exhibit the highest burden of HAP-induced stroke—diverges from previous research, which frequently emphasizes low-SDI regions as the most affected by air pollution exposure. To address this, we have revised the fifth paragraph of the "Discussion" section (originally beginning with "Significant differences exist in the stroke burden related to HAP across different regions and countries") to include a detailed discussion of this divergence. The revised paragraph now explains that: (1) in low-SDI regions, limited healthcare access and underreporting may lead to an underestimation of the stroke burden in the GBD 2021 dataset, masking its full extent; (2) middle-SDI regions, such as parts of East Asia and South Asia, face a unique dual burden from persistent household use of solid fuels and increasing ambient pollution due to rapid urbanization and industrialization, amplifying HAP-related stroke risk; and (3) unlike high-SDI regions, middle-SDI regions have not yet fully transitioned to clean energy, while potentially benefiting from better health data reporting compared to low-SDI regions, which may result in higher recorded burdens. This explanation highlights the complex relationship between socioeconomic development, exposure patterns, and data availability, positioning middle-SDI regions as a critical transitional phase of heightened vulnerability.

**Reviewer point #11:** Discussion: Some countries (e.g., Vietnam, Mongolia) experienced an increase in stroke burden due to HAP, while others (e.g., Estonia, Norway) showed a sharp decline. What policy or environmental factors might explain these differences?

**Author response #11:** We appreciate the reviewer's insightful comment regarding the stark differences in HAP-related stroke burden trends across countries. As noted in our findings, countries like Vietnam and Mongolia experienced significant increases in stroke burden due to HAP, while Estonia and Norway showed sharp declines. These disparities indeed warrant further discussion of underlying policy and environmental factors. In response to this valuable suggestion, we have expanded our discussion to include a detailed analysis of potential explanatory factors for these country-level variations. Specifically, we have added a new paragraph discussing how differences in energy transition policies, economic development trajectories, regulatory frameworks, geographic and climate conditions, and healthcare system development may contribute to these observed trends.

2. **Reviewer 1**

**Reviewer point #1:** The authors present a valuable study on the epidemiology of stroke attributed to household air pollution. Their findings indicate decreasing mortality, DALY, and YLL trends but an increasing YLD trend after age adjustment. However, these results may be significantly influenced by evolving treatment modalities that reduce both stroke-related mortality and morbidity, representing an important confounding factor. This limitation should be explicitly discussed.

**Author response #1:** We thank this comment. We agree that evolving treatment modalities for stroke could represent an important confounding factor influencing the observed trends of decreasing age-standardized mortality, DALYs, and YLLs, alongside an increasing YLDs trend. To address this, we have revised the manuscript by explicitly discussing this limitation in the Discussion section (Section 4), specifically in the paragraph addressing study limitations (the penultimate paragraph of the section). We have added a detailed explanation highlighting how advances in medical interventions—such as improved acute stroke care (e.g., thrombolysis and endovascular therapy), better secondary prevention strategies (e.g., antihypertensive and statin therapies), and enhanced rehabilitation programs—may have reduced mortality and severe disability while increasing the prevalence of individuals living with milder, long-term disabilities. We also note that these effects might be more pronounced in high-SDI regions, aligning with the regional disparities observed in our findings. This addition strengthens the interpretation of our results by acknowledging the potential impact of treatment advancements as a confounding factor.

**Reviewer point #2:** Additionally, in Figure 4, the YLD trend does not align with other indices specifically in high-middle SDI countries, which warrants further explanation. The study may also benefit from discussing potential future trends based on geographic distribution, such as SDI categories, to provide a more comprehensive perspective.

**Author response #2:**  We thank the reviewer for their insightful observation and valuable suggestion. Regarding the discrepancy in the YLD trend in high-middle SDI countries as depicted in Figure 4, we agree that this divergence from other indices—such as deaths, DALYs, and YLLs—merits further clarification. In high-middle SDI regions, the age-standardized YLD rate shows a less pronounced decline, or in some cases a slight increase, compared to the consistent downward trends observed for mortality, DALYs, and YLLs from 1990 to 2021. This pattern likely reflects the unique nature of YLDs as a measure of non-fatal disability, which may be influenced by factors such as improved survival rates following stroke events, increased detection and reporting of stroke-related disabilities, or a slower transition away from HAP exposure in these regions compared to high-SDI areas. High-middle SDI countries, often in a transitional phase of socioeconomic development, may experience a combination of persistent HAP exposure and enhanced healthcare access, leading to more individuals living with stroke-related disabilities rather than succumbing to fatal outcomes. We have added a brief explanation of this phenomenon in the revised "4. Discussion" section to clarify the observed trend and its potential drivers, while acknowledging that further investigation into regional health system dynamics and HAP exposure patterns could provide deeper insights.

Regarding the suggestion to discuss potential future trends based on geographic distribution, such as SDI categories, we appreciate the potential value this could add to the study’s perspective. However, we were unable to perform such projections due to the absence of population data stratified by SDI regions in the Global Burden of Disease (GBD) 2021 dataset or other accessible sources. The Nordpred model used for our projections relies on historical trends and population forecasts, but these are not disaggregated by SDI categories in a way that would allow us to reliably predict future burdens specific to each SDI level. This limitation prevented us from extending the analysis to SDI-stratified future trends.

As an alternative enhancement to provide a more comprehensive perspective, we have incorporated gender-stratified predictions into the manuscript, leveraging the available sex-specific data from the GBD 2021 dataset. In the revised "3.7 Future Burden of HAP-Induced Stroke" section, "4. Discussion" section, and "Abstract", we now present projections through 2040 that highlight the persistent gender disparity, with males projected to bear a higher absolute burden of deaths, YLDs, YLLs, and DALYs compared to females, despite declining ASRs for both sexes. This addition, supported by Figure 8 and Table S7, strengthens the study by addressing future trends in a stratified manner, focusing on sex as a key demographic factor influencing the stroke burden attributable to HAP. We believe this revision aligns with the reviewer’s intent to enrich the study’s forward-looking analysis, albeit through a different stratification lens due to the data constraints noted above.

**Reviewer point #3:** Finally, the authors should ensure that the image resolution is sufficient, as the current version is not fully understandable.

**Author response #3:**  We sincerely thank both the reviewer and the academic editor for highlighting the issue regarding the clarity and resolution of the figures in our initial submission. We fully acknowledge that the figures, as originally submitted, were not of sufficient quality, being too small and blurred, which likely impeded their readability and interpretation. We apologize for any inconvenience this may have caused during the review process.

To address this concern, we have taken the following steps in the revised manuscript:

1. Increased Resolution: All figures have been re-generated and updated to a higher resolution of 300 dpi, ensuring that the visual details are sharp and clear.
2. Adjusted Size: We have enlarged the dimensions of each figure to improve visibility of text, labels, data points, and map features, making them more legible without compromising the overall layout of the manuscript.
3. **Reviewer 2**

**Reviewer point #1:** This manuscript is novel and useful for the current literature, since it is debating on an association that has been newly described in literature. The addition of projection for 2040 by using Nordprod prediction model further approves the point.

**Author response #1:** We sincerely thank Reviewer 2 for their positive assessment of our manuscript and for recognizing its novelty and utility within the current literature. We are gratified that the reviewer finds value in our exploration of the association between household air pollution (HAP) and stroke burden, an area that is indeed gaining increasing attention but remains underexplored on a global scale with the latest data. The acknowledgment of our study’s contribution to this emerging field is greatly appreciated and aligns with our aim to provide a timely update and extension to prior research.

4. **Reviewer 3**

**Reviewer point #1:** The study provides a valuable update on the global stroke burden related to household air pollution (HAP), using the latest GBD 2021 data. However, while the projections to 2040 add a predictive aspect, it would be beneficial to clarify how this study substantially extends prior GBD-based research.

**Author response #1:** We sincerely thank Reviewer 3 for their positive evaluation of our study and for recognizing its value as an update on the global stroke burden attributable to HAP using the latest GBD 2021 data. We appreciate the opportunity to clarify how our work substantially extends prior GBD-based research, and we have revised the manuscript in the Introduction section and the Discussion section to address this point more explicitly.

**Reviewer point #2:** The manuscript is well-structured but dense with statistical details. Some sections, such as the methods and results, could benefit from improved readability by incorporating more subheadings.

**Author response #2:** We thank Reviewer 3 for their positive feedback on the manuscript’s structure and for their constructive suggestion to improve readability by incorporating more subheadings in the "Methods" and "Results" sections. We acknowledge that the dense statistical details in these sections, while comprehensive, could benefit from enhanced clarity and navigation to make the content more accessible to readers.

To address this comment, we have revised both sections by adding subheadings to break down the text into more manageable and logically distinct segments:

1. In the "Methods" section (Section 2), we introduced subheadings under each major subsection (2.1, 2.2, 2.3). For example, under "2.1 Data Source," we added "2.1.1 Data Collection," "2.1.2 Socio-demographic Index (SDI) and Regional Classification," and "2.1.3 Age Stratification" to separate data acquisition, classification criteria, and age assumptions. Similarly, under "2.3 Statistical Analysis," we included "2.3.1 Time Trend Analysis," "2.3.2 Cluster Analysis," "2.3.3 Correlation Analysis," and "2.3.4 Future Projections" to clearly delineate the analytical approaches (see revised Section 2).
2. In the "Results" section (Section 3), we added subheadings to distinguish between burden estimates and trends. For instance, under "3.1 The Burden of Stroke Attributable to Indoor Air Pollution and Its Temporal Trends," we included "3.1.1 Global Burden in 2021" and "3.1.2 Temporal Trends (1990-2021)." Under "3.5 Variation in HAP-Induced Stroke Burden by GBD Regions," we added "3.5.1 Regional Burden in 2021," "3.5.2 Regional Trends and Clustering," and "3.5.3 Correlation with SDI" to separate current data, trend analysis, and correlation findings (see revised Section 3).
